# Pediatric robotic‐assisted laparoscopic ureterocalycostomy: Salient tips and technical modifications for optimal repair

**DOI:** 10.1002/bco2.53

**Published:** 2020-11-14

**Authors:** B. L. Adamic, A. Lombardo, C. Andolfi, D. Hatcher, M. S. Gundeti

**Affiliations:** ^1^ Pediatric Urology Section of Urology Department of Surgery Comer Children’s Hospital Pritzker School of Medicine The University of Chicago Chicago IL USA; ^2^ Pritzker School of Medicine The University of Chicago Chicago IL USA; ^3^ Sharp Memorial Hospital San Diego CA USA

**Keywords:** pediatric, robotic, ureterocalicostomy, ureteropelvic junction obstruction

## Abstract

**Introduction:**

Ureterocalycostomy is a necessary option for renal salvage in cases where conventional reconstructions have failed or as a primary option in anatomic situations such as intrarenal pelvis, malrotated, or horseshoe kidney. The primary principle of this procedure is to allow for dependent drainage. Ureterocalycostomy is often difficult due to extensive scar tissue and may be complicated by bleeding in the setting of a normal functioning lower pole cortex, compared to thin renal cortex and poor renal function as seen in end‐spectrum of the obstruction. Identification of a dependent calyx and hemostasis can be difficult when there is a normal cortical thickness. Though the vascular control of hilum is an option, we suggest some simple tips to avoid this step and optimize surgical results. We present our experience and salient technical tips with pediatric robotic‐assisted laparoscopic ureterocalycostomy and provide a step‐by‐step video.

**Methods:**

Four patients underwent robotic‐assisted laparoscopic ureterocalycostomy between the years 2012 and 2016 by a single surgeon. Perioperative outcomes measured included operative time, hospital stay, pain relief, degree of hydronephrosis on postoperative ultrasound at 3 months, and renal scintigraphy as needed. We describe the operative procedure and provide tips on identifying a dependent lower pole calyx with flexible nephroscopy and needle puncture, the use of harmonic scalpel for incision of the lower pole cortex, and anastomosis by pre‐placement of interrupted sutures as the urothelium of the renal calyces is thin and friable.

**Results:**

Patients ranged in age between 11 months and 14 years old. Three of four patients had one prior pyeloplasty, and one patient had two prior pyeloplasties. Mean operative time (incision to closure) was 208 minutes. No Clavien‐Dindo 30‐day complications were encountered and no patients required blood transfusion. Anatomic success was reported in all patients with a mean follow‐up of 4.46 years; however, one patient ultimately required nephrectomy despite patent anastomosis, which would not drain due to a capacious pelvis.

**Conclusions:**

Robotic‐assisted laparoscopic ureterocalycostomy is feasible in re‐operative cases with extensive scaring and in patients with normal lower pole renal cortex. We offer tips to allow for safe and proficient performance of this procedure.

## 1 INTRODUCTION

Ureterocalycostomy is a necessary option in cases where conventional reconstructions have failed or as primary option in anatomic situations such as intrarenal pelvis, malrotated, or horseshoe kidney. In re‐operative cases, a scarred renal pelvis and significant peripelvic fibrosis is often encountered. The ureter is often engulfed in scar tissue and may be difficult to identify. Ureterocalycostomy is useful in patients with extensive peripelvic scarring, allowing for dependent drainage, and may compensate for lack of adequate ureteral length. Laparoscopic and robotic‐assisted laparoscopic ureterocalycostomy has a good track record of success in the adult literature.^1^, ^2^, ^3^, ^4^, ^5^ As this procedure is infrequently performed, there is a paucity of literature regarding robotic ureterocalycostomy in the pediatric population.^6^, ^7^ We report our experience with robotic ureterocalycostomy of a single surgeon and describe salient tips for successful repair, especially in patients with ample renal cortex where identification of a dependent cortex may be difficult and bleeding may occur during lower pole nephrectomy.

### 1.1 Preoperative work up

All patients undergo a renal ultrasound and a MAG3 lasix renogram prior to operative intervention. In patients who have required percutaneous nephrostomy tubes, an antegrade nephrostogram can be used to better delineate anatomic obstruction. MR Urography can be an alternative tool in the preoperative setting in the complex re‐operative patient. MRU provides functional and anatomic information, but often requires sedation for children and is costly, without much utility as a renal scan can provide similar functional information. An on‐table retrograde pyelogram is performed to delineate the anatomy of the ureter, UPJ, and assess length of scarring.

### 1.2 Surgical technique

A retrograde pyelogram is performed noting ureteral and UPJ anatomy, length of stricture, and a ureteral stent is placed when feasible. We use a 5 French 22‐32 cm multiloop ureteral stent ®Cook. Otherwise an open‐ended catheter can be placed at the level of obstruction.

The patient is placed in a lateral position with all pressure points padded. An 8/12 mm robotic umbilical camera port is placed using the open Hasson technique. The two working arms robotic 8 mm ports are placed under direct visualization—one in the midline in the epigastric region below the xiphoid and the other equidistant from the umbilicus to the anterior superior iliac spine. In children younger than 5 years old, all ports are placed midline. The 5 mm assistant port can be placed in the midline suprapubic, or at the contralateral midclavicular line. In patients with right‐sided disease, often an additional 5mm liver retractor assistant port is made in the midline.

The colon is reflected medially using a combination of blunt and sharp dissection along the white line of Toldt. The transmesocolic approach on the left side is discouraged as the exposure is limited. The ureter is identified in the retroperitoneum and dissected proximally. The proximal ureter and ureteropelvic junction are further dissected as able. In cases of extensive fibrosis at the UPJ, the UPJ can be tied off and proximal ureter transected. The vascular hilum is not routinely dissected.

The ureter is ligated from the ureteropelvic junction and a stay stitch can be placed at the UPJ. In cases of substantial thinning of the renal cortex, a simple incision can be made onto a dependent calyx.

In patients with ample renal cortex, it may be difficult to identify a calyx. A flexible nephroscope can be advanced through the renal pelvis to aid in identification of an appropriate calyx. (Salient Tips 1) An angiocath can be used to puncture the calyx “towards the light” in patients with a thick renal cortex. In patients with a thin renal cortex, the nephroscope light can be easily identified and a dependent calyx nephrotomy is easily performed. Bleeding is often encountered when incising the renal cortex. A harmonic scalpel can be useful when creating this nephrotomy. (Salient Tips 2) Approximately a 1 cm wedge is removed and the urothelium of the lower pole calyx can be identified.

The use of a stay suture on the lower pole of the kidney and/or hitch stitch can allow for ease of reconstruction due to the mobility of the lower pole. (Salient Tips 3).

The previously placed stent is reinserted into the dependent lower pole calyx. The ureter is spatulated and anastomosed to the lower pole with 4‐0 polydioxanone (PDS) in an interrupted fashion. As the calyceal urothelium is delicate and sutures may easily tear, we suggest pre‐placing all sutures first on the calyx. (Salient Tips 4) Next the interrupted anastomotic sutures are placed on the spatulated ureter and tied down one at a time. The defect in the renal pelvis is closed with 4‐0 PDS suture.

Drain placement is recommended to both diagnose and control a urine leak especially in cases with a precarious anastomosis. (Salient Tips 5) These patients often have extensive scaring which raises concern regarding the healing of the anastomosis. Drain creatinine is only sent if the output is high, and is removed after 48‐72 hours.

A 2‐0 polyglactin (Vicryl) suture is used to close fascia of all port sites under vision.

The foley can be removed the following morning. The drain can be removed thereafter if there are no clinical signs of urine leak. The indwelling ureteral stent is removed 4‐6 weeks postoperatively.

**Table:** 

Salient tips for robotic‐assisted ureterocalycostomy
Flexible nephroscopy to identify dependent calyx and minimize nephrotomy to preserve nephrons Use of harmonic scalpel when creating nephrotomy to decrease bleeding, without need to clamp hilum Use of stay suture or hitch stitch for reconstruction due to mobility of the lower pole of kidney Pre‐placement of anastomotic sutures on calyx as these easily tear Drain placement is recommended to diagnose and control a urine leak

## 2 METHODS

We retrospectively identified four patients who underwent robotic‐assisted laparoscopic ureterocalycostomy performed by a single surgeon between the years 2012 and 2016. Outcomes measured included preoperative characteristics such as T1/2, split renal function, renal cortical thickness, age, body mass index (BMI), intraoperative findings, estimated blood loss, and length of hospital stay. The stent was removed at 4‐6 weeks postoperatively and all patients underwent renal ultrasound after 3 months. MAG3 renal scintigraphy is performed 3‐6 months postoperatively if indicated.

## 3 RESULTS

A total of four patients underwent robotic‐assisted laparoscopic ureterocalycostomy. Table 1 displays patient characteristics. The age range was from 11 months to 14 years old, and half of patients were male. The average BMI was 18.63. Three of four patients had one prior pyeloplasty, and one patient had two prior pyeloplasties. Three patients required a percutaneous nephrostomy tube be placed prior to surgery. A Nephrostogram confirmed recurrent UPJO with no or little passage of contrast into the proximal ureter. Only one patient had substantial cortical thinning (<5 mm) while the remaining patients had normal cortical thickness.

**TABLE 1:** Patient characteristics

Patient	Age	Gender	BMI	Pre‐Op Hydro Grade	History	Months since prior operation
1	11 months	Female	20.5	3	Solitary kidney (MCDK), open pyeloplasty age 5 months, anuria requiring PCN after stent removal	6
2	14 years	Male	22.2	4	Prior robotic pyeloplasty c/b urinoma. Pre‐op PCN, flank pain	10
3	4 years	Male	14.46	4	Laparoscopic and robotic pyeloplasty at age 3 years	15, 8
4	14 years	Female	17.36	2	Prior robotic pyeloplasty, pre‐op PCN	3

Table 2 displays perioperative characteristics and outcomes. Mean operative time was 208 minutes from skin incision to skin closure and includes time necessary to dock the robot. Intraoperatively, two patients were noted to have an intrarenal pelvis and three patients had extensive scarring. Mean EBL was 27.5cc and no patients required a blood transfusion. Mean hospital stay was 3.5 days.

**TABLE 2:** Operative details and outcomes

Patient	Laterality	Prior UPJO intervention	Intra‐op findings	EBL (mL)	Pre‐op T1/2	Pre‐op % function	Pre‐op cortical thickness (mm)	Post‐op T1/2	Post‐op % function	Post‐op cortical thickness (mm)
1	R	1	High insertion, malrotation	15	>20	NA	6.2		NA	no thinning
2	L	1	Extensive scarring, capacious renal pelvis	10	NA	44	5.4		46	4.1
3	R	2	Extensive scarring	10	24	49	3.5	17.3	49	4.5
4	R	1	Extensive scarring, Intrarenal pelvis	75	16	57	6.1	5.9	54	6.5

No patients had an early complication within 30 days postoperatively. Postoperative renal ultrasound revealed improvement in hydronephrosis in two patients and stability in the other two. Split renal function was stable and patients who underwent MAG3 had an improvement in T1/2. A MAG3 was obtained for patient 3 due to a UTI and persistent (but stable) hydronephrosis. Patient 4 had a MAG 3 due to flank pain 5 months postoperatively, who was ultimately diagnosed with chronic constipation and abdominal pain.

At a mean follow‐up of 4.46 years, all patients had an anatomically successful ureterocalycostomy. One patient (patient 2) had persistent pain despite replacement of a ureteral stent (post operative day 108). He ultimately underwent a nephrectomy just over a year after ureterocalycostomy, despite a patent anastomosis, due to poor drainage from a large capacious renal pelvis. One patient had recurrent UTIs for 3 years postoperatively due to bowel bladder dysfunction, which resolved with biofeedback and bowel regimen.

## 4 DISCUSSION

Ureterocalycostomy is often used as a salvage operation for patients who have had previously failed initial pyeloplasty due to peripelvic scarring and fibrosis. Other indications include intraoperative findings such as intrarenal pelvis, inadequate viable ureteral length to perform a tension‐free anastomosis, malrotated, or horseshoe kidney. Preoperative planning is aided by obtaining detailed anatomic information via antegrade nephrostogram or retrograde pyelography. Renal function should be assessed with nuclear renography or MRU.

Surgical principals to be adhered to include adequate nephrotomy or guillotine amputation of the lower pole of the kidney, preservation of ureteral blood supply, widely spatulated ureter, tension free, mucosa to mucosa anastomosis, and stenting. In patients with a thicker renal cortex, it can be challenging to find the dependent calyx. A flexible nephroscope can be advanced through the renal pelvis in this situation to aid in identification of an appropriate calyx. When performing the lower pole nephrectomy for patients with preserved cortical thickness, robust bleeding can be encountered. Vascular control by clamping the hilum is an option, however this has disadvantages including warm ischemia time, possibility of inadvertent hilar injury, and increased operative time. We have found that the harmonic scalpel can be used to minimize blood loss when creating the nephrotomy/lower pole nephrectomy. No patients in our case series required blood transfusion. The harmonic scalpel transmits a large amount of energy and heat to tissues which is advantageous for hemostasis. When using the harmonic scalpel, one must take caution near the collecting system, as significant tissue necrosis can lead to postoperative urine leak due to anastomotic breakdown. As only a nephrotomy is made, maximally sparing the lower pole parenchyma, we have had success without requiring clamping of the hilum. Significant bleeding can be encountered during the cortical dissection, especially if a guillotine amputation is planned, therefore, hilar control may be necessary. The use of a stay suture on the lower pole of the kidney and/or hitch stitch can allow for ease of reconstruction. We recommend pre‐placing interrupted anastomotic sutures on the calyx first, as the calyceal urothelium is delicate and sutures may easily tear. A separate suture is not used to evert the calyceal edge. Then the interrupted sutures can be placed on the spatulated ureter and tied. A drain is routinely placed. These tips allow for proficiency during this operation.

One of the most feared and frequent complication following ureterocalycostomy is recurrent obstruction. Success of UC has varied, with adult literature 60%‐77%.^1^, ^8^ and pediatric literature with a 70%‐90% success rate or greater.^6^, ^7^, ^9^, ^10^, ^11^ Success between open and minimally invasive techniques have not been directly compared, but appear to be similar. Failure rates correlate with poor preoperative GFR and renal cortical thickness of <5 mm.^1^ Mean time to failure in a large series was 5.5 months with majority of patients failing within a year.^1^ Authors recommend guillotine lower pole nephrectomy in order to reduce anastomotic stricture rates.^12^ We have had success with a lower pole nephrotomy. The flexible nephroscopy guided needle puncture onto a dependent calyx allows for appropriate exposure and wide anastomosis without lower pole nephrectomy. We believe that guillotine amputation of the lower pole leads to unnecessary loss of nephrons in patients who may have a compromised kidney. With a median follow‐up of 4.46 years, we believe this technique prevents long‐term obstruction.

In two patients who underwent MAG3 renal scintigraphy postoperatively, both showed a decrease in the T1/2 of the affected moiety. One patient in this series did proceed to nephrectomy due to chronic flank pain and a poorly draining, capacious renal pelvis despite patency of the ureterocalycostomy as seen on multiple retrograde pyelograms. He required stenting 3.5 months postoperatively. Hydronephrosis persisted despite stenting and the patient continued to have flank pain. He was managed with ureteral stenting for 1 year prior to ultimately undergoing a nephrectomy. We suspect this kidney was poorly functioning, and that the MAG3 finding of a 46% split renal function appeared to be an incorrect estimation, as the area of interest was over estimated by including the collecting system. Cortical thickness is a surrogate of renal function and DMSA after percutaneous nephrostomy placement can additionally be used to obtain an accurate estimate of the split renal function. This patient had a poor cortical thickness and was ultimately unable to generate adequate hydrostatic pressure to allow for drainage. On an antegrade nephrostogram, his renal pelvis capacious, and was able to accommodate 500 mL prior to drainage. In patients with a poorly functioning renal unit, an upfront nephrectomy can be offered. Our practice is to preserve nephrons especially in the pediatric population, therefore, despite a poorly functioning kidney, attempt was made for salvage.

Recurrent urinary tract infection (UTI) is another common complication following ureterocalycostomy which occurs in up to 30% of patients.^1^ Only one patient in our series had recurrent UTIs postoperatively which successfully resolved with a bowel regimen and biofeedback.

Other reported complications such as urinary leak and bleeding requiring transfusion were not observed in our cohort. A drain is routinely left in place in order to manage these complications.

In most cases, this operation may salvage the kidney and avoid nephrectomy. In pediatric patients with poorly functioning moieties (split function <20%), surgical intervention to improve drainage may allow modest renal recovery with low rates of failure (3%).^13^ The combined assessment of renal ultrasound and DMSA with a decompressed kidney may offer a more accurate depiction of renal function. Alternative salvage operations include ileal ureter, buccal mucosal grafting, appendiceal interposition, and auto transplantation (with pyelocystostomy) which can add morbidity and uncertainty in regard to long‐term outcomes. Buccal mucosal grafting in upper tract reconstruction is a novel technique which has been recently popularized with high rates of success in an adult population.^14^ This may be an appropriate salvage maneuver in children who fail pyeloplasty, however long‐term outcomes should be established before applying this technique to a vulnerable pediatric population. Other considerations of the buccal mucosal graft utilization in children include the potential morbidity of facial deformity and contracture. Additionally, the length of buccal mucosa graft able to be harvested from a pediatric population may be inadequate for complex upper tract reconstruction.

## 5 CONCLUSION

Robotic‐assisted laparoscopic ureterocalycostomy in children is feasible in re‐operative cases with extensive scaring and in patients with normal lower pole renal cortex. We offer tips to allow for safe and proficient performance of this procedure.

## CONFLICT OF INTEREST

Dr Mohan Gundeti is co‐director of the NARUS course. There are no other conflict of interest.
